# The effects of storage temperature and duration of blood samples on DNA and RNA qualities

**DOI:** 10.1371/journal.pone.0184692

**Published:** 2017-09-19

**Authors:** Lien-Hung Huang, Pei-Hsien Lin, Kuo-Wang Tsai, Liang-Jen Wang, Ying-Hsien Huang, Ho-Chang Kuo, Sung-Chou Li

**Affiliations:** 1 Genomics and Proteomics Core Laboratory, Department of Medical Research, Kaohsiung Chang Gung Memorial Hospital and Chang Gung University College of Medicine, Kaohsiung, Taiwan; 2 Department of Biological Sciences, National Sun Yat-sen University, Kaohsiung, Taiwan; 3 Department of Medical Education and Research, Kaohsiung Veterans General Hospital, Kaohsiung, Taiwan; 4 Department of Child and Adolescent Psychiatry, Kaohsiung Chang Gung Memorial Hospital and Chang Gung University College of Medicine, Kaohsiung, Taiwan; 5 Kawasaki Disease Center, Kaohsiung Chang Gung Memorial Hospital, Kaohsiung, Taiwan; 6 Department of Pediatrics, Kaohsiung Chang Gung Memorial Hospital and Chang Gung University College of Medicine, Kaohsiung, Taiwan; 7 Department of Pediatrics, Chiayi Chang Gung Memorial Hospital, Chiayi, Taiwan; Universitat de Lleida, SPAIN

## Abstract

DNA and RNA samples from blood are the common examination target for non-invasive physical tests and/or biomedical studies. Since high-quality DNA and RNA samples guarantee the correctness of these tests and/or studies, we investigated the effects of storage temperature and storage duration of whole blood on DNA and RNA qualities. Subjects were enrolled to donate blood samples which were stored for different durations and at different temperatures, followed by the examinations on RNA quality, qPCR, DNA quality and DNA methylation. For RNA, we observed obvious quality decline with storage duration longer than 24 hours. Storage at low temperature does not keep RNA samples from degradation. And, storing whole blood samples in freezer dramatically damage RNA. For DNA, quality decline was not observed even with storage duration for 15 days. However, DNA methylation significantly altered with storage duration longer than three days. Storage duration within 24 hours is critical for collecting high-quality RNA samples for next-generation sequencing (NGS) assays (RIN≧8). If microarray assays are expected (RIN≧7), storage duration within 32 hours is acceptable. Although DNA is resistant within 15 days when kept in whole blood, DNA quantity dramatically decreases owing to WBC lysis. In addition, duration for more than three days significantly alter DNA methylation status, globally and locally. Our result provides a reference for dealing with blood samples.

## Introduction

Circulation system is responsible for the transportation of oxygen, nutrition, water, waste and metabolite around human body. In animal, the concentration of these transported chemicals within circulation system tend to keep a balanced homeostatic state. Therefore, examining the concentration of these chemicals in blood may indicate one's physical conditions, especially for metabolic conditions. In animal, lymphocytes in circulation system function as protectors, conducting many immune activities to protect animals from external damage, chemical or biological. Therefore, examining the patterns of lymphocytes in blood may reflect one's immune conditions, especially for inflammatory conditions.

Because of high correlation with physical and immune conditions, DNA, RNA and protein samples from blood, especially from white blood cell (WBC) in most cases, are the common examination targets for non-invasive physical tests and/or biomedical studies. Margaritelis and colleagues determined tissue oxidative stress by measuring redox biomarkers (enzyme, metabolite and vitamin) in blood [1]. Kuo *et al* identified susceptibility genes associated with coronary artery aneurysm formation in Kawasaki disease by analyzing WBC DNA [2]. Tang and colleagues observed IL-27 decline in progressive multiple sclerosis by examining PBMC RNA [3]. Moreover, many studies showed that it was applicable to use blood miRNAs to predict disease [4–9]. Low-quality samples usually leads to inconsistent results. Therefore, the correctness of physical test and the success of biomedical study largely depend on high-quality DNA and RNA samples from blood.

Since the qualities of DAN and RNA samples from blood are critical to the result of physical test and biomedical study, we are interested in how to collect high-quality DNA and RNA samples from blood. In most cases, blood sample collection and further nucleic acid extraction from blood are conducted by different people at different places. So, the storage duration and storage temperature, from blood collection to nucleic acid extraction, are critical to high-quality DNA and RNA samples. Although the fresher the better is the golden rule when dealing with clinical samples, including liquid biopsies, we are interested in how much storage duration and storage temperature of blood affect the qualities of DNA and RNA. Therefore, we enrolled subjects and collected blood samples. These blood samples were stored for different durations and at different temperatures, followed by extracting nucleic acids and evaluating the qualities and quantities of DNA and RNA samples.

## Methods

### Ethics, consent, permission and clinical blood sample collection

This study has been approved by the Institutional Review Board of Chang Gung Memorial Hospital. All subjects signed the informed consent. Blood was collected using scalp vein set (NIPRO, Osaka, Japan), vacutainer^®^ One-Use holder and Vacutainer^®^ Blood Collection Tubes (with heparin, REF367874, BD, New Jersey, USA). All samples were categorized according to storage duration and storage temperature. After specified storage duration at specific temperature, blood samples were treated with dextran to induce RBC aggregation. The supernatant was then harvested and RBC lysis buffer was added into supernatant. After centrifugation, total WBCs were harvested from the pellets.

### DNA and RNA extraction

For RNA extraction, the harvested WBCs from 5ml blood were lysed with lysis/binding buffer and RNA isolation was conducted with mirVana^TM^ miRNA Isolation Kit (Ambion, CA, USA), according to the manufacturer’s protocol. Next, the isolated RNA samples were eluted with 50 μl elution buffer. For DNA extraction, WBCs from 2ml blood were suspend with 1ml PBS and DNA isolation was conducted with QIAamp^®^ DNA Blood Mini Kit (QIAGEN, CA, USA), according to the manufacturer’s protocol. Next, the isolated DNA samples were eluted with 100 μl elution buffer. The extracted DNA and RNA samples were quantified with NanoDrop 2000 spectrophotometer (Thermo scientific, MA, USA).

### Determination of DNA and RNA quality

RNA quality was assessed with the RNA integrity number (RIN) by Agilent 2100 Bioanalyzer (Agilent, CA, USA) with RNA 6000 Nano Kit, according to the manufacturer’s protocol. DNA quality was assessed by Agilent 2100 Bioanalyzer (Agilent, CA, USA) with DNA 1000 Kit.

### Real-time quantitative polymerase chain reaction

For 18S rRNA, GAPDH and IP-10 quantification, 0.5 μg RNAs were reverse transcribed to cDNA by High-Capacity cDNA Reverse Transcription Kit (Applied Biosystems, CA, USA). The cDNA reverse transcription master mix was prepared as follow: 2 μl 10X RT buffer, 0.8 μl 25X dNTP mix (100mM), 2 μl 10X RT random primers, 1 μl reverse transcriptase, 1 μl RNase inhibitor, 4.2 μl nuclease-free water and 10 μl RNA sample. The thermal-cycling condition was as follow: 10 min at 25°C, 120 min at 37°C, 5 min at 85°C and hold at 4°C. Next, the quantification job was conducted with Fast SYBR^®^ Green Master Mix and StepOnePlus ^TM^ System (Applied Biosystems). The sequences of the primers used were as follow:

18S: forward primer (5’- GTAACCCGTTGAACCCCATT -3’) and reverse primer (5’- CCATCCAATCGGTAGTAGCG -3’);

GAPDH: forward primer (5'-ATGGGGAAGGTGAAGGTCG-3') and reverse primer (5'-GGGGTCATTGATGGCAACAAT-3');

IP-10: forward primer (5’-TCTAGAACTGTACGCTGTACC-3’) and reverse primer (5’-GATTCAGACATCTCTTCTCACC-3’). The real-time PCR master mix was prepared as follow: 10 μl 2X fast SYBR green master mix, 1 μl forward primer (10 μM), 1 μl reverse primer (10 μM), 1 μl cDNA and 7 μl nuclease-free water. The default PCR thermal-cycling condition was as follow: 20 sec at 95°C and 40 cycles of 3 sec at 95°C and 30 sec at 60°C.

For RNU6B (U6) quantification, the RNAs were reverse transcribed to cDNA by TaqMan MicroRNA Reverse Transcription Kit (Applied Biosystems) and TaqMan^®^ MicroRNA Assays (RNU6B, PN4440887). The RT master mix was prepared as follow: 0.3 μl 100Mm dNTPs, 3 μl reverse transcriptase, 1.5 μl reverse transcription buffer, 0.19 μl RNase inhibitor, 6 μl primer, 1.01 μl nuclease-free water and 3 μl RNA. The RNU6B expression was quantified using TaqMan^®^ Fast Universal PCR Master Mix (2✕), No AmpErase^®^ UNG (Applied Biosystems) and StepOnePlus ^TM^ System. The real-time PCR master mix was prepared as follow: 7.5 μl 2X master mix, 0.8 μl probe, 6.7 μl nuclease-free water and 5 μl cDNA. The default PCR thermal-cycling condition was as follow: 20 sec at 95°C and 40 cycles of 3 sec at 95°C and 30 sec at 60°C.

### Pyrosequencing

DNA methylation was assessed by pyrosequencing. As suggested by the manufacturer’s protocol, 0.5 μg genomic DNA was bisulfite modified using EZ DNA Methylation-Lightning ^TM^ Kit (Zymo Research, Irvine, USA). Next, the bisulfite modified DNAs were eluted with 10 μl elution buffer, followed by PCR amplification with PyroMark PCR Kit (Qiagen). The PCR reagent was prepared as follow: 12.5 μl 2X master mix, 0.5 μl forward primer (10 μM), 0.5 μl reverse primer (10 μM), 5 μl 5X Q solution, 2.5 μl 10X coralload concentrate, 1.5 μl MgCl2, 0.5 μl nuclease-free water and 2 μl bisulfite modified DNA. After PCR reaction, we ran gel assay to make sure the success of PCR reaction and the correctness of the size of PCR product. Following amplification, the methylation percentage of CpG marker cg08298591 was assessed with PyroMark Q24 (Qiagen). The primer sequences are as follow: forward primer (5'-GTAGTAGTTAGAGTGAGTGGTTTAGAGTT-3'), reverse primer (Biotin 5'-TACCAAAAACATAACATTTCAAACAACTT-3') and sequencing primer (5'-GGTTTTTTATTTATTTAAAGGTAGG-3').

### Dot blotting assay

DNA samples (100ug) were added to denaturation buffer (0.4mM NaOH and 10mM EDTA) and denatured at 100°C for 10min. Then, samples were chilled on ice for 5min and applied to a positive charged nylon membrane (Roche, Basel, Switzerland). The membrane was UV cross-linked and dried for 1hr at 70°C. Membranes were probed with anti-5mC antibody (1:1000, Cat. No. 61255, Active Motif) at 4°C overnight. Subsequently, membranes were probed with either a rabbit or mouse IgG antibody conjugated to HRP for 1 hour at room temperature. After three times of wash with PBS-Tween, immunoreactive dot were detected using an ECL kit (Amersham Pharmacia Biotech). Finally, the membranes were stained with Methylene Blue as loading control.

### Statistical analyses and data availability

In this study, statistical analyses were conducted with Microsoft Excel. All quatitative raw data for plotting each figure and sub-figure is available in S1 Table.

## Results

### Quality examination on RNA samples

**Fig 1:**
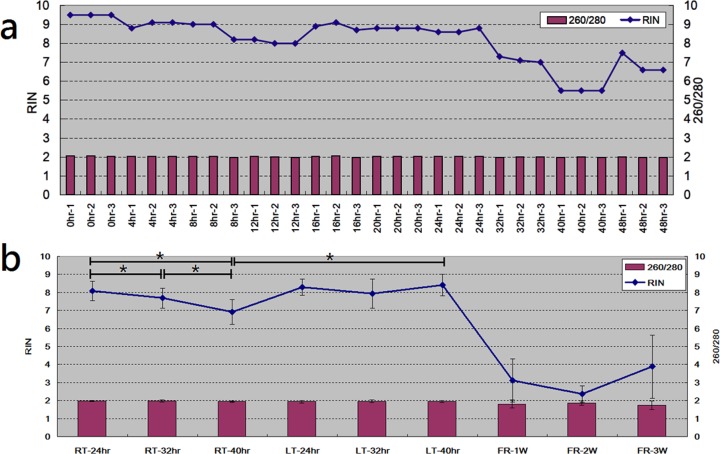
Examinations of RNA quality on samples with different storage temperatures and storage durations. RNA quality was evaluated based on RIN value measured with ABI Bioanalyzer. 260/280 value was also determined to exclude contamination. * denoted significance by t-test. (a) X axis represented different storage durations of whole blood. The samples in 0hr set were subject to WBC enrichment and RNA extraction immediately after blood collection. All blood samples were collected from one single subject and the storage temperature is 24°C. The RIN value curve showed a decline at the duration point of 32hr, demonstrating RNA decayed after 24hr. (b) RT, LT and FR demonstrated that blood samples were stored at room temperature (24°C), low temperature (4°C) and at freezer (-80°C), respectively. The samples in FR set were stored at freezer immediately after blood collection. After specified durations, they were kept at room temperature for two hours to return to liquid form and to reach up to the balanced 24°C. Consistent with Fig 1A, RNA decayed after 24hr. Low temperature protected RNA from degrading at the duration of 40hr. Each subject contributed three independent blood samples. As a result, for each condition, 30 blood samples were collected from 10 subjects (N = 30). Data was presented as mean±SD. All quatitative raw data for plotting each figure and sub-figure is available in S1 Table.

To examine how much time can blood samples be stored without a significant decline in RNA quality, we first drew blood samples from one single subject and stored the blood samples at room temperature (RT, 24°C) with different storage durations, followed by WBC enrichment and RNA extraction (see *Materials and Methods*section). The collected RNA samples were then subject to RNA integrity number (RIN) and 260/280 examination with Bioanalyzer and NanoDrop spectrophotometer, respectively. As shown in Fig 1A, the 260/280 ratio remained consistent in different sets, demonstrating the high purity of RNA samples. The RIN value remained ≧8.0 in all samples with the duration sets ≦24 hours. In the 32hr duration sets, the RIN values remained ≧7.0. RIN ≧8.0 and RIN ≧7.0 are two common quality criteria for RNA samples being analyzed with next-generation sequencing (NGS) [10] and microarray [11], respectively. According to Fig 1A, if NGS and microarray experiments are expected, the blood samples can be stored, at room temperature, no longer than 24 and 32 hours, respectively.

From the preliminary result, 32hr is a clear duration point at which RIN value started to decline. To derive a more robust and unbiased conclusion, we conducted more detailed assays with more samples. We drew 27 independent blood samples from each of the 10 subjects and repeated the analyses at three storage durations (24, 32 and 40hr) and at three temperatures (RT, LT and FR), where RT, LT and FR individually denoted room temperature (24°C), low temperature (4°C) and frozen (-80°C). As a result, 270 blood tubes were collected (3 replications * 3 temperatures * 3 durations * 10 subjects)., Fig 1B demonstrated similar result with Fig 1A. In addition, when samples were stored at low temperature, the RNA quality remained without gradual declines. For the frozen samples, the RNA quality declined dramatically. To provide the evidence to RNA quality decline, the analysis outputs of Bioanalyzer were illustrated in S1 Fig.

### Determination of qPCR Ct values on RNA samples

**Fig 2:**
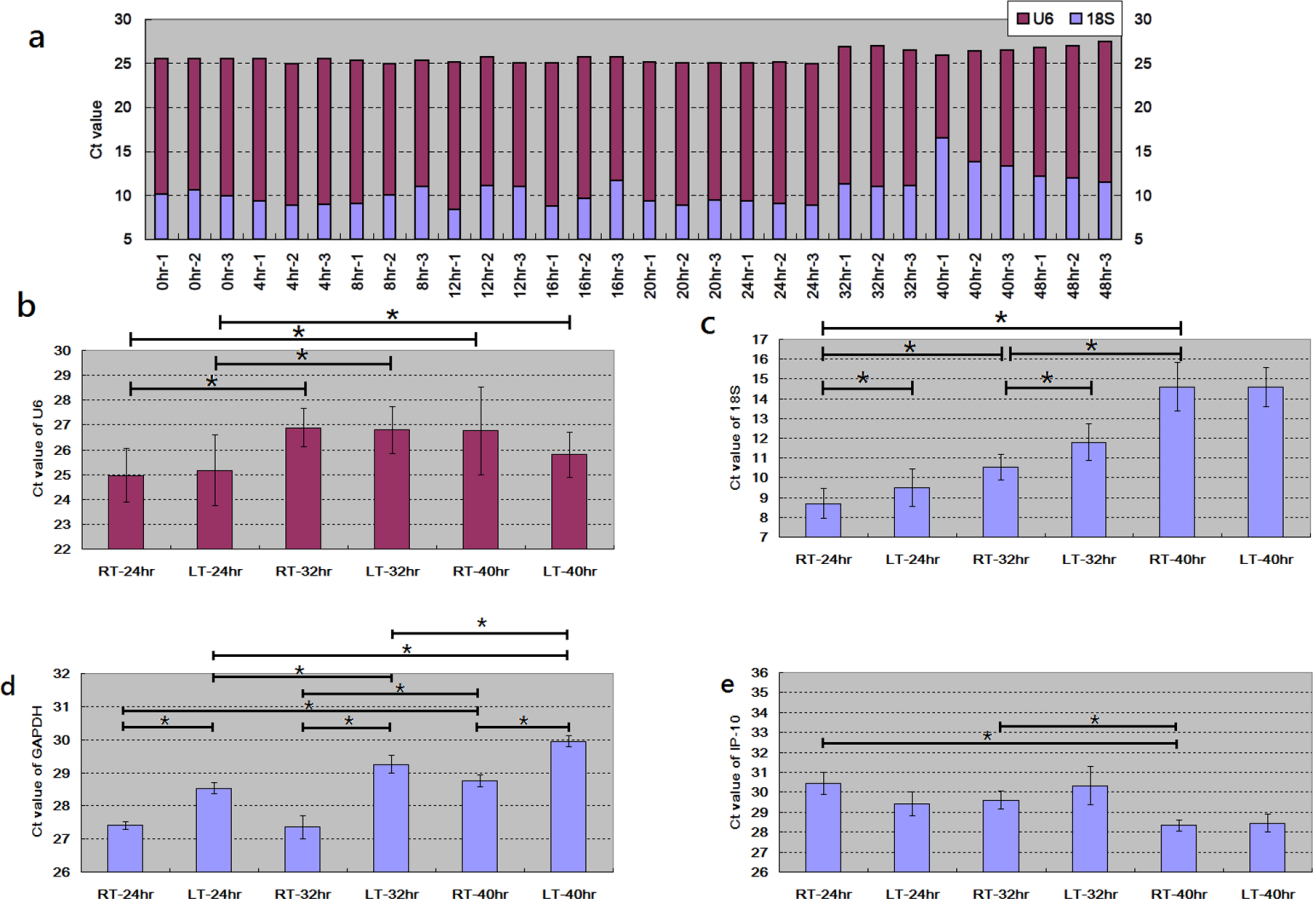
Examinations of qPCR Ct value on samples with different storage temperatures and storage durations. * denoted significance by t-test. (a) All blood samples were collected from one single subject and stored at 24°C. The Ct values of U6 and 18S significantly rose at the duration of 32hr. (b,c,d,e) Thirty blood samples were collected from 10 subjects (N = 30) for each condition. Consistent with Fig 1, with longer duration, more RNA decayed and less U6, 18S and GAPDH mRNA detected. Compared with room temperature, low temperature did not protect genes from further degradation. Data was presented as mean±SD.

RNA samples are usually used for gene expression profiling with real-time quantitative polymerase chain reaction (qPCR). Therefore, in addition to RNA quality examination, we also investigated the expression abundances on three common internal control genes with qPCR assay. The same RNA samples presented in Fig 1A were further subject to qPCR assay to determine the Ct values of 18S and U6, where the former and the latter are common internal control genes for protein-coding gene and miRNAs, respectively. For the samples with storage duration ≦ 24 hours, the Ct values of U6 were pretty consistent and were approximately 25 (mead = 25.28, stand deviation = 0.29, Fig 2A). When the samples were stored longer than 24 hours, the Ct values of U6 increased up to approximately 27 with little variations. For the ≦24 hr samples, although the Ct values of 18S varied, most of them were less than 10 (mean = 9.61, stand deviation = 0.96). For the >24 hr samples, the Ct values of 18S dramatically varied.

Fig 2A brought the conclusion, similar with Fig 1A, that 24hr is a golden duration for high-quality RNA sample. To derive a more robust and unbiased result, the samples presented in Fig 1B were also subject to qPCR assay on 18S, U6 and GAPDH. Fig 2B, 2C and 2D demonstrated that with longer storage duration, the Ct values of U6, 18S and GAPDH increased. Fig 2B also showed that at the duration point 24, 32 and 40 hour, storage at low temperature made no difference on the Ct values of U6 compared from the one at room temperature. However, for 18S and GAPDH low temperature contributed to significantly higher Ct values at the duration point 24 and 32 hour. Such results implied that low temperature storage was also harmful to the expression consistency of 18S and GAPDH but harmless to the one of U6. In summary, storage of whole blood in low temperature is not recommended.

qPCR assay on WBCs samples is often conducted to observe the expression profiles of immune-related genes. In addition to internal control genes, we also examined one chemokine gene, Interferon gamma-induced protein 10 (IP-10, also named CXCL-10). IP-10 is induced by interferon gamma and involved in many inflammatory diseases such as chronic inflammatory arthritis, autoimmune diseases, type 1 diabetes, cardiovascular disease, psoriasis…etc [12–16]. Fig 2E showed that higher expression abundance of IP-10 is expected in longer storage durations. When stored in tube, blood cells are quarantined from continuous supply of oxygen, nutrition and so on, which brought stress to blood cells. Although with larger variations than 18S and GAPDH, the expression of IP-10 is activated by stress in spite of RNA degradation.

qPCR assay was conducted to determine the relative abundances of gene expression transcripts. Therefore, the less degraded the RNA samples are, the more consistent Ct values the RNA samples have. Although Fig 1B showed that storing blood samples at low temperature, compared with at room one, prevented RNA from degradation, the Ct values of 18S and GAPDH still rose even stored at low temperature. In spite of the contradiction, our finding suggested that the blood samples should be stored for no longer than 24 hours at room temperature to obtain the best quality of RNA samples.

### Quality and quantity examination on DNA samples

In addition to RNA, DNA from blood is also usually examined for SNP calling [2], InDel identification [17], methylation profiling [18] and so on. To examine how much time can blood samples be stored without a significant decline in DNA quality, we first drew blood samples from one single subject, storing blood samples at room temperature and at different durations. Then, we harvested WBCs by RBC lysis, followed by collecting DNA samples. S2 Fig showed that the DNA samples with different durations aggregated with the size larger than 10K base pair. So, even stored for 15 days, DNA samples did not significantly degrade into small fragments. The same conclusion was also confirmed with Bioanalyzer DNA 1000 chip assay (data not shown).

**Fig 3:**
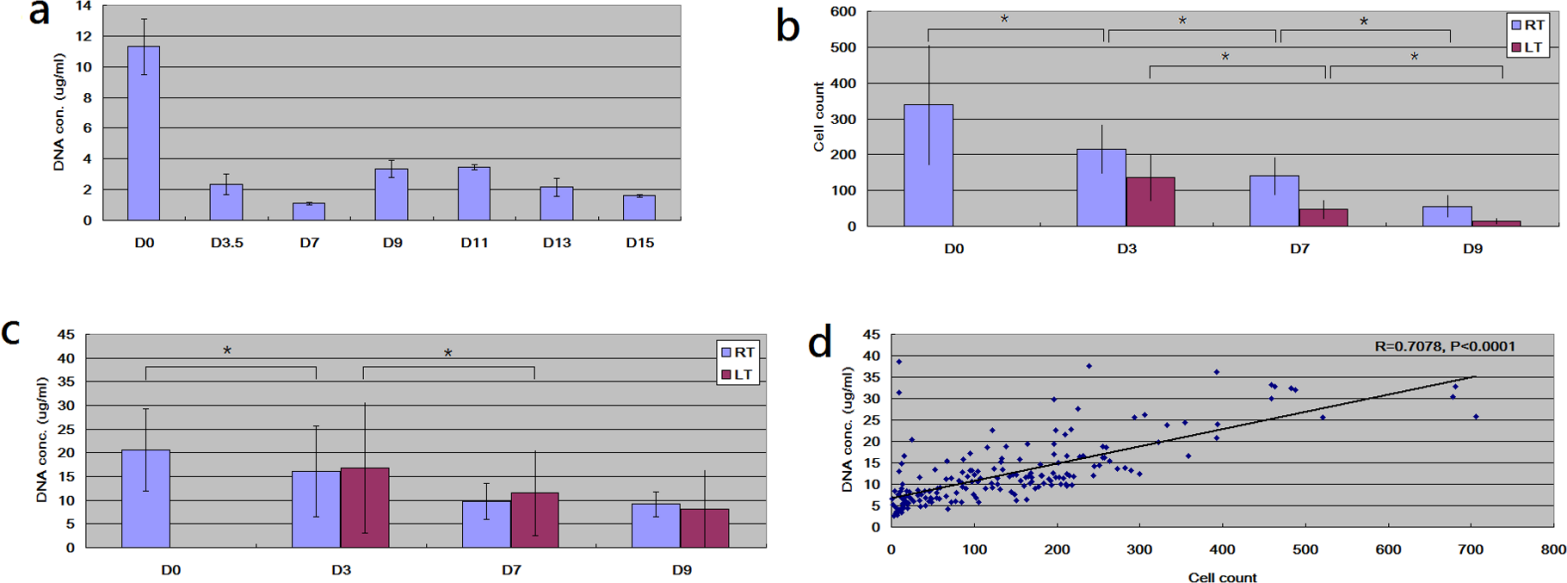
Examinations of WBC count and DNA concentration on samples with different storage temperature and storage duration. * denoted significance by t-test. All data was presented as mean±SD. (a) X axis represented different storage durations of whole blood. For example, D0 denoted that whole blood was processed immediately after collected; D7 denoted that whole blood was stored for 7 days before further processing. All blood samples were collected from one single subject and stored at 24°C. For each duration point, three blood samples were collected (N = 3). (b,c) With longer duration, more WBCs lysed and less DNA was collected from cell pellets. Thirty blood samples were collected from 10 subjects (N = 30). (d) WBC number and DNA concentration were highly correlated, showing that decreased DNA collection was resulted from WBC lysis. Scatter plot was generated from 199 data points (7*30 = 210, excluding several failed samples).

Since it seemed that DNA quality did not significantly decline with long storage duration, we turned to investigate DNA quantity, measuring the DNA concentration on the samples. Fig 3A demonstrated significant declines in DNA concentration for storage duration after 3.5 days. So far, our result showed that, with long storage duration, the quality of DNA did not decline but the concentration of DNA did. For an unbiased answer to this question, we drew 21 blood samples from each of the 10 subjects and repeated the analyses at four storage durations and at three temperatures. As a result, 210 blood tubes were collected (3 replications * 7 conditions * 10 subjects). After collecting DNA samples, we counted the numbers of WBC. Fig 3B showed that longer storage duration resulted in fewer WBC counts at both room and low temperature. In addition, for the same storage duration, low temperature also led to fewer WBC counts (not illustrated in Fig 3B for simplicity). Such decline in WBC count should be resulted from cell lysis triggered by stress.

Further, we investigated DNA concentration. As shown in Fig 3C, at room and low temperature, longer storage duration also caused lower DNA concentrations. To interrogate the relationship between WBC count and DNA concentration, we plotted WBC count and DNA concentration on XY plan, calculating the correlation coefficient. Fig 3D showed that the two factors were highly correlated. Storing blood samples in tube stops the continuous supply of oxygen, nutrition and so on, which brought stress to blood cells. These stresses triggered blood cell lysis, lowering down the number of harvested WBC. In this study, we collected DNA and RNA from WBC pellet. So, the more WBCs lysed, the less DNA concentration.

### Methylation examination on DNA samples

**Fig 4:**
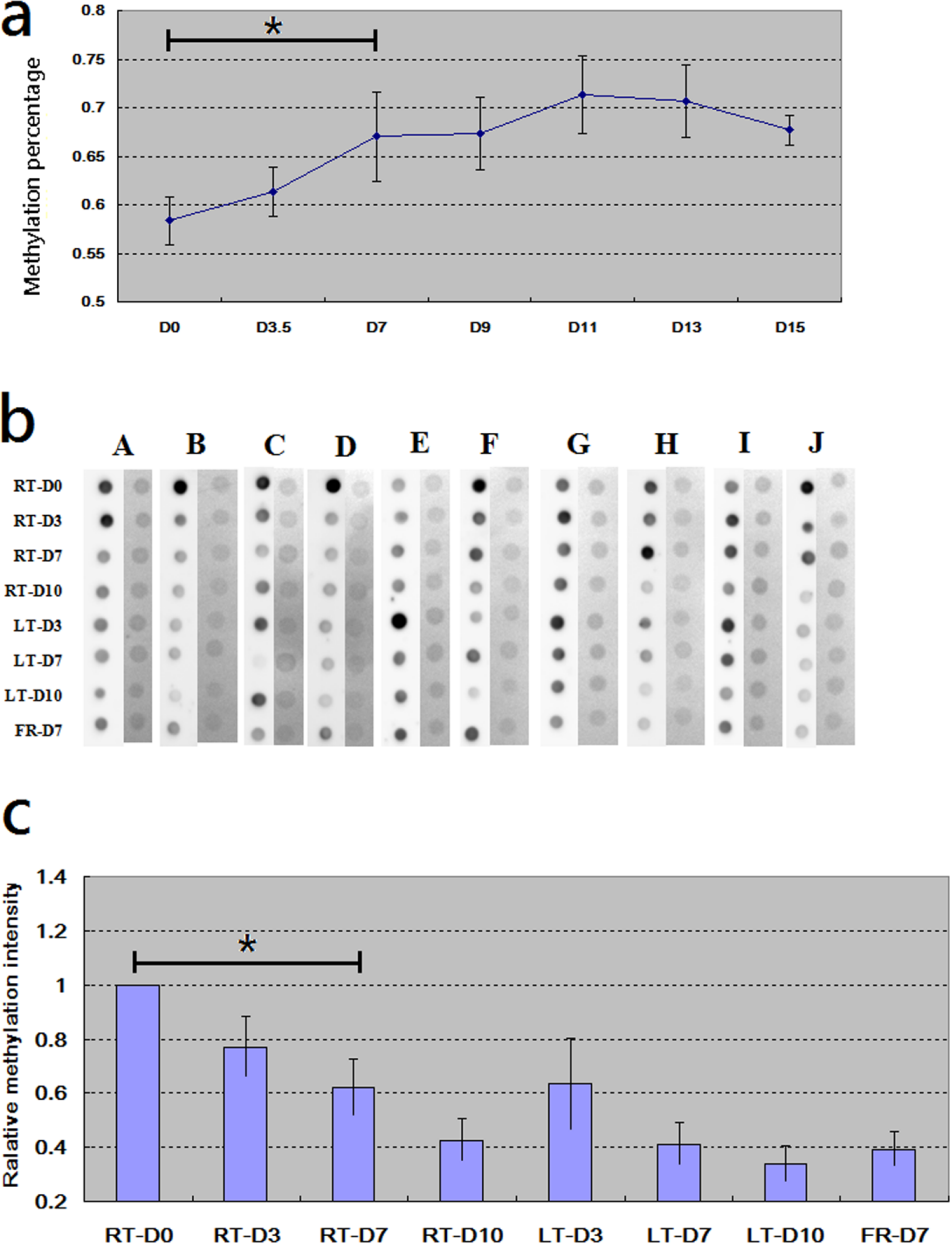
Examinations on DNA methylation. We investigated whether the variation on DNA methylation could be observed owing to different storage temperature and storage duration. * denoted significance by t-test. (a) CpG marker cg08298591 is located at CCL22. We determined its methylation percentage on samples. All blood samples were collected from one single subject and stored at 24°C. For each duration point, three blood samples were collected (N = 3). Compared with fresh condition (D0), cg08298591 was differentially methylated at the duration point D7 (*P*<0.05). Data was presented as mean±SD. (b) We conducted dot blot assay to evaluate global DNA methylation. DNA samples from ten subjects (A, B, C and so on) were collected (N = 10), forming different combinations of temperature and duration. For the samples from each subject, the left column denoted the methylation intensities of different samples and the right column denoted the corresponding loading control. (c) The methylation intensities in Fig 4B were normalized based on the corresponding loading controls and plotted. Data was presented as mean±SEM. With longer duration, no matter at room or low temperature, global methylation intensities decreased.

DNA samples from WBC are also usually used for evaluating DNA methylation [19, 20]. Therefore, in addition to DNA quantity, we are also interested in whether storage duration and storage temperature affect DNA methylation. To answer this question, we used pyro-sequencing facility (Pyromark Q24) to specifically evaluate the methylation status of CpG marker cg08298591 (located at the promoter region of CCL22). Fig 4A demonstrated that the methylation status of cg08298591 at D7 was significantly higher than it was at D0 (*P*<0.05). In addition, with longer duration, the methylation status of cg08298591 gradually increased. So, storage duration DID affect DNA methylation at consistent temperature.

The conclusion of Fig 4A was acquired by examining only one specific CpG marker on only one subject. For a more robust and unbiased conclusion, we conducted DNA methylation dot blood assay [21] at different duration and temperature on more subjects. As shown in Fig 4B, the fresh DNA samples have the highest global DNA methylation status. With longer duration, the global DNA methylation status gradually decreased at both room and low temperature (Fig 4C), including frozen samples.

## Discussion

Previous studies tried to develop the protocol for dry DNA storage and shipment [22]. In addition, several studies focused on how storage conditions affected the stability of serum proteins [23, 24]. Although Lv *et al* conducted a study to evaluate mRNA change with different time courses, they worked on rat's spleen tissue [25]. Blood not only reflects the oxidative stress of tissues [1] but also is an indicator of metabolic status [26]. Therefore, DNA and RNA samples from blood, especially from WBC in most cases, are the common examination target for physical tests and/or biomedical studies. The correctness of physical test and the success of biomedical study largely depend on high-quality DNA and RNA samples from blood.

In this study, we used heparin-containing tubes to collect blood samples. It was reported that heparin was usually used as an anticoagulant and could interfere with PCR reaction, producing aberrant results in qPCR or PCR assays [27, 28]. However, it was also reported that such interference by heparin contamination took place mostly on the RNA samples from serum or plasma, not on the one from WBC if heparin was clearly washed out [29, 30]. To examine whether heparin contamination interfere with PCR reaction on WBC-enveloped RNA samples and to test the applicability of our protocols, we conducted a comparison assay, where we collected blood samples, from six subjects, with six heparin-containing tubes and six EDTA- containing ones (Vacutainer^®^ Blood Collection Tubes; REF367835, BD, New Jersey, USA). Next, we conducted the same RNA enrichment procedures immediately after blood drawing, followed by qPCR reactions. For 18S, the heparin and EDTA qPCR Ct values were 9.65±0.70 (mean±S.D.) and 10.06±0.61, respectively; For GAPDH, they were individually 17.27±0.86 and 18.97±1.64; For IP-10, 25.41±0.95 and 26.42±0.92 were detected; For U6, the two qPCR Ct values were 25.47±0.11 vs. 25.54±0.18, respectively. In summary, none of the four genes had significantly different (p<0.05) Ct values by heparin or EDTA. So, heparin did not contaminate the RNA samples from WBC and did not interfere with PCR reaction according to our protocols.

The fresher the better is the golden rule when dealing with clinical samples, including liquid biopsies. In most hospitals, blood samples were collected by the specially-assigned staff rather than the people conducting physical tests and/or biomedical studies. So, the storage duration and storage temperature, from blood collection to nucleic acid extraction, are critical to high-quality DNA and RNA samples. As a result, storage duration and storage temperature of blood samples largely influence the correctness of physical test and the success of biomedical study.

IP-10 is a protein-coding gene and usually participates in inflammatory diseases, served as a pro-inflammatory chemokine. It has been reported that hypoxia conditions in murine lung and brain caused higher expression level of IP-10 [31, 32]. Storing blood in tube stops the continuous supply of oxygen, unavoidably triggering a hypoxia condition to blood cells. Therefore, we would like to know whether the level of IP-10 in the blood cells varied with different storage durations and storage temperature. Fig 2E indicated that longer storage durations made WBC produce more IP-10. Therefore, consistent with previous studies, longer duration caused severer hypoxia and higher levels of IP-10.

According to our result, although DNA is still resistant within 15 days when kept in whole blood, DNA quantity dramatically decreases owing to WBC lysis. In addition, DNA methylation status may alter with storage duration of whole blood. Actually, few studies have focused this issue. Vilahur and colleagues found that storing placenta tissues at room temperature for one, two, six and 24 hours brought small changes on global DNA methylation status [33]. In this study, dramatic declines of global DNA methylation status were observed when storing whole blood for more than 3 days. Although a global decline was observed, DNA methylation status of specific CpG marker (cg08298591) increased with storage duration extended. In other words, the globally overall pattern and the locally specific one are in the reverse direction. This conflict between global and local patterns deserves further attentions. The factors regulating DNA methylation alteration within blood also need further investigation as well.

## Conclusions

In this study, we investigated how much storage duration and storage temperature of whole blood affect the qualities of DNA and RNA. For RNA, storage duration within 24 hours is critical for collecting high-quality RNA samples for NGS assays (RIN≧8). If microarray assays are expected (RIN≧7), storage duration within 32 hours is acceptable. In addition, storage at low temperature does not keeping RNA samples from degradation. Most important of all, storing whole blood samples in freezer dramatically damage RNA samples. Although DNA is resistant within 15 days when kept in whole blood, DNA quantity dramatically decreases owing to WBC lysis. In addition, duration for more than three days significantly alter DNA methylation status, globally and locally. Our findings provide a reference for collecting high-quality DNA and RNA from blood samples.

## Supporting information

S1 Fig The output illustration of Bioanalyzer RNA 6000 Nano assay. We conducted Bioanalyzer RNA 6000 Nano assay to examine RNA quality. Bioanalyzer calculated RIN value according the relative intensities of 18S and 28S to marker. As shown in this Fig, the samples with high RIN value have relatively strong and clear 18S and 28S peaks. And, the samples with low RIN value have relatively weak and ambiguous 18S and 28S peaks.
(TIF)

S2 Fig The gel illustration of DNA quality. We conducted DNA electrophoresis with 0.5% argarose gel. The gel illustration showed that DNA samples were resistant without breaking down into small fragments even with the storage duration of 15 days.
(TIF)

S1 Table The raw data for plotting figures. The raw data for plotting each figure and sub–figure is available in S1 Table. These digits are RIN value, 260/280 value, qPCR Ct value, WBC count, WBC concentration or methylation percentage. Empty columns denote failed experiments.
(XLS)
