# Supplementary figures and images for: The effects of storage temperature and duration of blood samples on DNA and RNA qualities
Source: PLoS One. 2017 Sep 19;12(9):e0184692. doi: 10.1371/journal.pone.0184692 (PMC5604973; doi:10.1371/journal.pone.0184692)

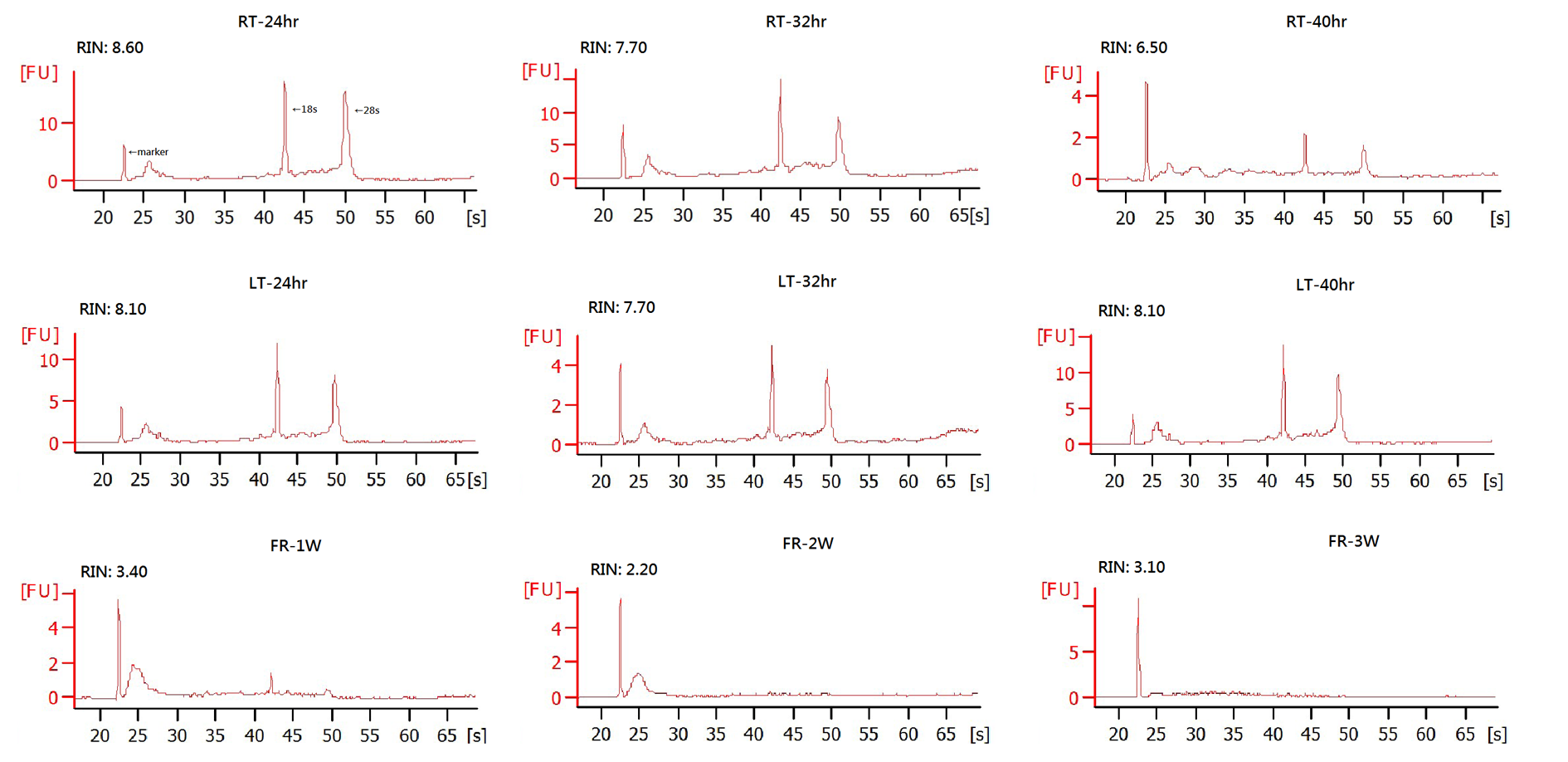

Supplement: S1 Fig — We conducted Bioanalyzer RNA 6000 Nano assay to examine RNA quality. Bioanalyzer calculated RIN value according the relative intensities of 18S and 28S to marker. As shown in this Fig, the samples with high RIN value have relatively strong and clear 18S and 28S peaks. And, the samples with low RIN value have relatively weak and ambiguous 18S and 28S peaks. (TIF) [file pone.0184692.s001.tif]

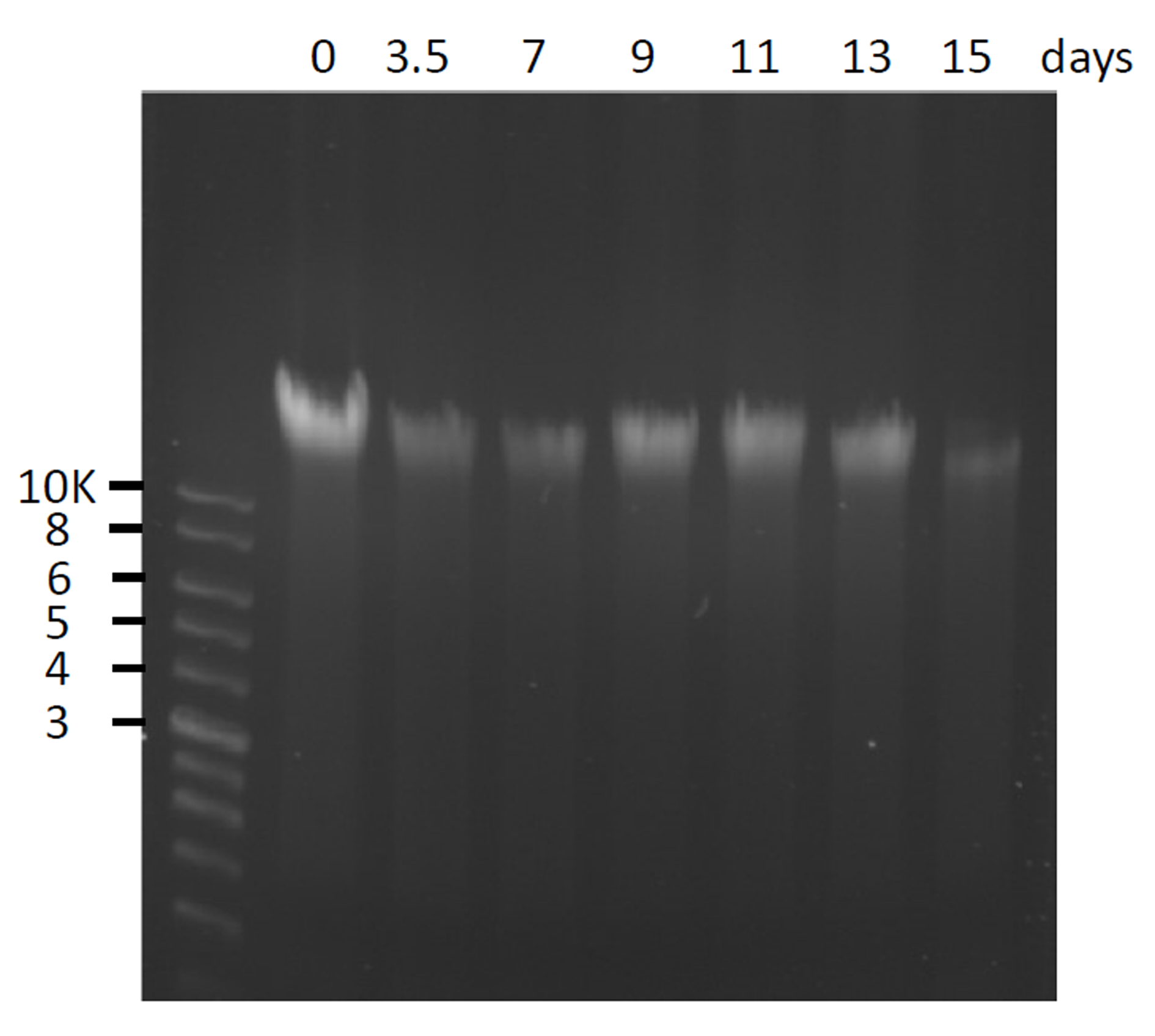

Supplement: S2 Fig — We conducted DNA electrophoresis with 0.5% argarose gel. The gel illustration showed that DNA samples were resistant without breaking down into small fragments even with the storage duration of 15 days. (TIF) [file pone.0184692.s002.tif]
